# Versatile and Marvelous Potentials of Polydeoxyribonucleotide for Tissue Engineering and Regeneration

**DOI:** 10.34133/bmr.0183

**Published:** 2025-04-14

**Authors:** Nuri Oh, Juyoung Hwang, Moon Sung Kang, Chung-Yul Yoo, Minseok Kwak, Dong-Wook Han

**Affiliations:** ^1^ Department of Chemistry and Biology, Korea Science Academy of Korea Advanced Institute of Science and Technology, Busan 47162, Republic of Korea.; ^2^ Department of Chemistry, Pukyong National University, Busan 48513, Republic of Korea.; ^3^ Smart Gym-Based Translational Research Center for Active Senior’s Healthcare, Pukyong National University, Busan 48513, Republic of Korea.; ^4^ Ajou Energy Science Research Center, Ajou University, Suwon 16499, Republic of Korea.; ^5^ Research Institute of Mechanical Technology, Pusan National University, Busan 46241, Republic of Korea.; ^6^ Department of Energy Systems Research and Chemistry, Ajou University, Suwon 16499, Republic of Korea.; ^7^ Industry 4.0 Convergence Bionics Engineering, Pukyong National University, Busan 48513, Republic of Korea.; ^8^ Department of Cogno-Mechatronics Engineering, Pusan National University, Busan 46241, Republic of Korea.; ^9^ Institute of Nano-Bio Convergence, Pusan National University, Busan 46241, Republic of Korea.

## Abstract

Over the past decade, substantial focus has been placed on polydeoxyribonucleotide (PDRN) due to its promising pharmacological properties, making it a valuable candidate for tissue engineering applications. Accordingly, this paper aims to review and summarize the latest experimental research on PDRN in the context of tissue engineering and regeneration. The unique biochemical mechanisms of PDRN to promote cellular behavior and regeneration are summarized. We categorize commonly utilized PDRN-based tissue engineering fields as neuromuscular tissues, diabetic wound or skin, and bone regeneration. At the same time, we explore scaffold strategies for integrating PDRN into bioceramics, polymers, and cell/tissue-derived materials, along with its combination with photo/electromodulation techniques. Furthermore, we discuss potential opportunities and challenges in translating PDRN-based approaches into clinical practice. We expect future interdisciplinary research and clinical trials to evaluate the long-term efficacy and safety of PDRN while emphasizing standardization and quality control to ensure its consistency and effectiveness in regenerative applications.

## Introduction

Human tissues suffer permanent damage from aging, trauma, infections, and diseases. While natural healing processes are often sufficient, severe cases may surpass the body’s capacity to fully restore structure and function. Traditional methods, such as bioinert implants and biological grafts (e.g., xenografts, autografts, and allografts), have been used to address these issues, but they involve intrinsic limitations such as mechanical mismatches, corrosion and wear behavior, and lowered host tissue integration. Meanwhile, biological grafts have disadvantages, including donor site morbidity, supply shortages, and immune reactions. In line with this, tissue engineering approaches have emerged as promising alternatives. Tissue-engineering-based scaffolds particularly provide appropriate characteristics such as affordable microstructures, controllable mechanical properties, biodegradability, nonimmunogenicity, and noncytotoxicity. A key characteristic of tissue engineering scaffolds is their ability to regulate cellular behaviors and fates by integrating bioactive cues. These biological cues, such as proteins, peptides, growth factors, biopolymers, and various chemicals, can trigger specific signaling pathways to enhance cell growth and maturation, leading to the development of functional tissues. However, in terms of clinical application, the effects of proteins are often shorter than desired and require very high, nonphysiological doses [1]. Moreover, while growth factors have been extensively tested in rodents, translating these findings to humans has proven challenging, with many uncertainties remaining about the appropriate dosing for effective treatment [2].

Polydeoxyribonucleotide (PDRN) is a bioactive form of polynucleotides (PNs), with molecular weights between 50 and 1,500 kDa, primarily sourced and refined from the sperm cells of trout (*Oncorhynchus mykiss*) or chum salmon (*Oncorhynchus keta*) [3]. PDRN consists of a linear polymer chain of deoxyribonucleotides, with purine and pyrimidine nucleotides as its building blocks. Through extraction and purification methods, over 95% pure substance can be obtained [4]. Sperm cells are the ideal source for extracting highly purified deoxyribonucleic acid (DNA), minimizing the presence of impurities such as peptides, proteins, and lipids, which help prevent immune reactions [5]. Furthermore, studies have shown many positive therapeutic effects of PDRN, such as angiogenesis, osteogenic differentiation, collagen synthesis, and anti-inflammatory effects, leading to its potential applications in tissue regeneration fields.

**Fig. 1.**
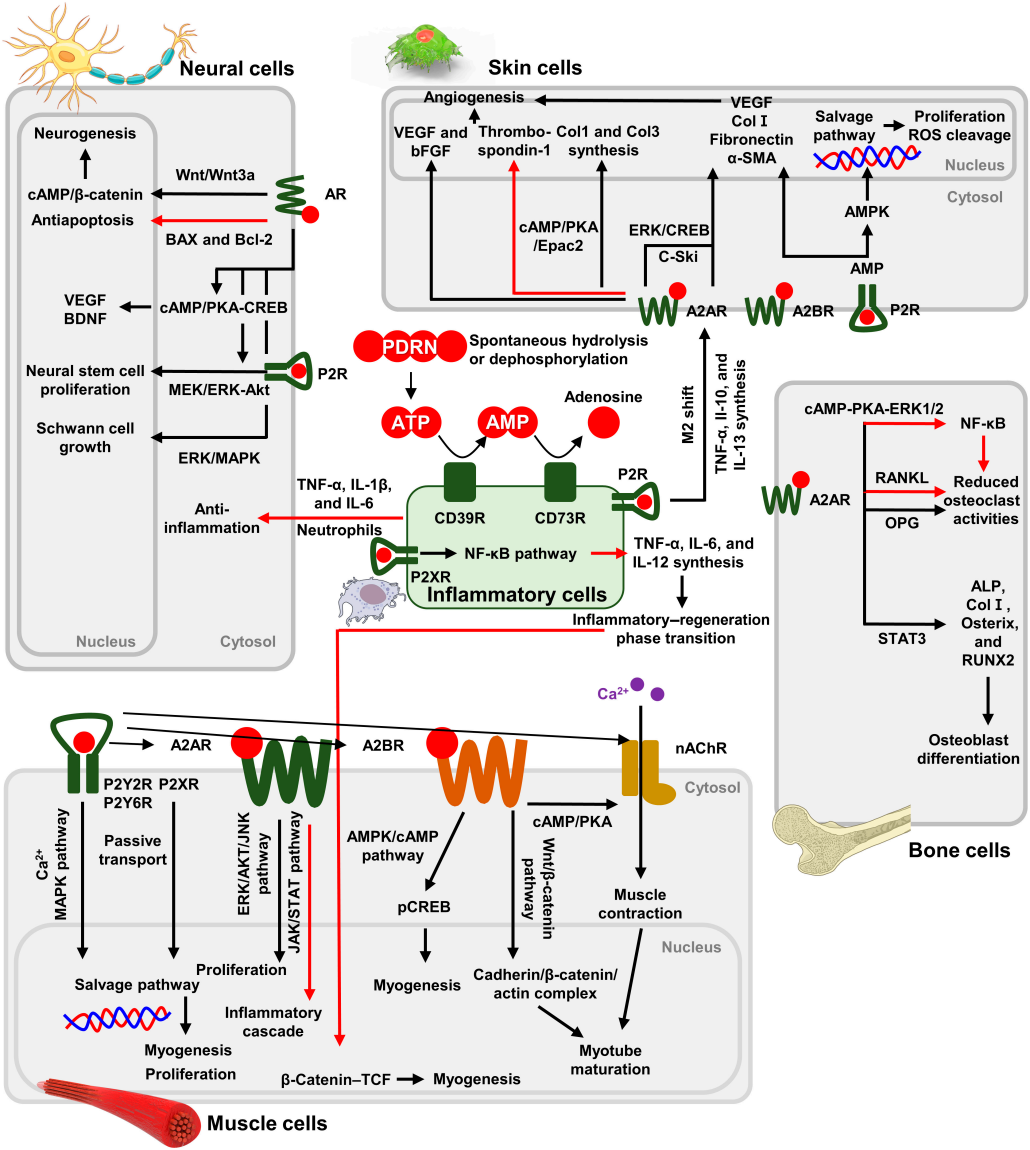
Schematic diagram of the representative signal pathways of polydeoxyribonucleotide (PDRN) for tissue regeneration. A2AR, adenosine A2A receptor; A2BR, adenosine A2B receptor; ALP, alkaline phosphatase; α-SMA, α-smooth muscle actin; AMPK, AMP-activated protein kinase; AMP, adenosine monophosphate; AR, androgen receptor; ATP, adenosine triphosphate; BAX, Bcl-2-associated X protein; Bcl-2, B-cell lymphoma 2; BDNF, brain-derived neurotrophic factor; bFGF, basic fibroblast growth factor; cAMP, cyclic adenosine monophosphate; Col I, type I collagen; Col3, type III collagen; CREB, cAMP response element-binding protein; Epac2, exchange protein directly activated by cAMP 2; ERK, extracellular signal-regulated kinase; IL-1β, interleukin-1 beta; IL-6, interleukin-6; IL-10, interleukin-10; IL-12, interleukin-12; IL-13, interleukin-13; JAK, Janus kinase; JNK, c-Jun N-terminal kinase; MAPK, mitogen-activated protein kinase; MEK, mitogen-activated protein kinase kinase; nAChR, nicotinic acetylcholine receptor; NF-κB, nuclear factor kappa B; OPG, osteoprotegerin; P2R, purinergic receptor; P2XR, purinergic P2X receptor; P2Y2R, purinergic P2Y2 receptor; P2Y6R, purinergic P2Y6 receptor; pCREB, phosphorylated CREB; PKA, protein kinase A; RANKL, receptor activator of nuclear factor κB ligand; ROS, reactive oxygen species; RUNX2, Runt-related transcription factor 2; STAT3, signal transducer and activator of transcription 3; TCF, T-cell factor; TNF-α, tumor necrosis factor-alpha; VEGF, vascular endothelial growth factor.

In this review, we introduce the distinctive properties of PDRN and the underlying biochemical mechanism of how PDRN promotes cellular behaviors and tissue regeneration (Fig. 1). Subsequently, we introduce recently highlighted studies on PDRN for tissue regeneration, categorizing its applications in neuromuscular tissues, skin, diabetic wounds, and bones. Various materials and methods, including bioceramics, polymers, cell/tissue-derived materials, and photo/electromodulations, were combined with PDRN to prepare tissue engineering scaffolds and grafts.

## Underlying Mechanism of PDRN for Tissue Regeneration

### Adenosine as a key component for tissue regeneration

PDRN is a linear polymer chain of deoxyribonucleotides with purine and pyrimidine nucleotides. Studies have found that the bioactive effects of PDRN mainly come from adenosine, a nucleoside composed of deoxyribose sugar and adenine (i.e., purine base) [2,6–8]. Adenosine is produced either through the spontaneous hydrolysis and dephosphorylation of adenosine triphosphate (ATP), where CD39 converts ATP to adenosine monophosphate (AMP), followed by CD73 dephosphorylating AMP to adenosine [9,10]. Adenosine modulates cell and tissue functions by mainly interacting with the purinergic P2X and P2Y receptors (P2XR and P2YR), adenosine A2A and A2B receptors (A2AR and A2BR), nicotinic acetylcholine receptor (nAChR), which subsequently stimulate the proliferation, differentiation, maturation, and anti-inflammatory pathways [11]. In addition, these multiple functions of adenosine have led to the development and commercialization of adenosine-based drugs. PDRN also modulates inflammatory reactions by inhibiting the nuclear factor kappa B (NF-κB) pathway and activating the Wnt/β-catenin pathway similar to the mechanism of adenosine, demonstrating its roles in immunomodulation in tissue regeneration [12]. In the following subsections, the representative signal pathways of PDRN for tissue regeneration are discussed with detailed biomolecular mechanisms.

### Proliferation-inducing effects of PDRN

The degradation process and specific signaling pathways of PDRN for proliferation, immune modulation, and differentiation to specific lineages (i.e., myogenesis, neurogenesis, wound healing, and osteogenesis) are summarized. PDRN is cleaved by active cell membrane enzymes, providing an abundant source for deoxynucleotides and deoxynucleosides [2]. Guizzardi et al. demonstrated that PDRN significantly stimulates the proliferation of human osteoblasts, showing a maximum increase of 21% after 6 d in vitro. The addition of 3,7-dimethyl-1-propargylxanthine (DMPX, a purinergic A2AR inhibitor) to PDRN-treated cells induced a 42.9% reduction in cell proliferation after 6 d, compared to that of only-PDRN-treated cells, suggesting that the A2AR pathway is not the only route but plays an important role in PDRN-induced cell proliferation. Gessi et al. [13] showed that the activation of A2AR by CGS21680 (agonist) promotes proliferation in the A549 lung, MRMT-1 breast, and A375 melanoma cancer cell lines. Increased phosphorylation levels were observed in the subsequent signaling cascades, including phospholipase C (PLC), protein kinase C-delta (PKC-δ), extracellular signal-regulated kinase 1/2 (ERK1/2), c-Jun N-terminal kinase 1/2 (JNK1/2), and AKT kinases. The addition of selective agonists (TP455 and ZM 241385) confirmed that A2AR engagement is critical for these cell proliferation pathways. A study by Jo et al. [14] showed the proliferative effects of A2AR pathways. In this study, PDRN treatment in an in vitro ischemia/reperfusion injury model showed anti-inflammatory effects by increasing the expression of anti-inflammatory genes (e.g., PTPN6 and RAC2) and reducing pro-inflammatory cytokines (e.g., colony stimulating factor 1 [CSF1] and interleukin-6 [IL-6]) through down-regulation of the Janus kinase/signal transducer and activator of transcription (JAK/STAT) pathway. PDRN acted as an A2AR agonist, which increased suppressor of cytokine signaling 3 (SOCS3) expression, further suppressing inflammatory cascades within the JAK/STAT pathway.

Besides the A2AR pathways, the P2XR and salvage pathways are also well-known mechanisms that enhance cell proliferation. Cui et al. [15] demonstrated that the external ATP increases DNA synthesis and enhances the affinities of proliferative growth factors through P2XR, by activating a mitogen-activated protein kinase (MAPK) pathway. Applying ATP directly to Sca-1^+^ cells led to an increased Ca^2+^ concentration, stimulating a significant increase in proliferation rates. In this process, ATP-induced proliferation primarily involves purinergic P2Y2 and P2Y6 receptors (P2Y2R and P2Y6R) through the activation of the P38–MAPK pathway. Therefore, overdosed extracellular ATP can target the P2Y2R- and P2Y6R-mediated Ca^2+^ and MAPK pathways, which can significantly promote cell proliferation. On the other hand, the salvage pathway is a cellular mechanism that recycles bases and nucleosides from the breakdown of DNA and ribonucleic acid (RNA), converting them back into nucleotides for DNA synthesis. PDRN and exogenous purine are known to induce this pathway, aiding in cell proliferation and tissue restoration, as observed in various cell types [5,16]. In a clinical trial by Lazzarotto et al. [17], PDRN eye drops demonstrated clear therapeutic effects, significantly promoting corneal healing following photorefractive keratectomy. By 3 d post-surgery, 77% of PDRN-treated corneas had completely re-epithelialized, compared to 61% in the standard therapy group. PDRN-treated eyes also showed a lower corneal evaluation score by day 3, indicating faster healing. In cases where patients served as their own controls (i.e., one eye treated with PDRN, the other with placebo), healing rates aligned with overall results, supporting PDRN’s effect on promoting consistent re-epithelialization.

### Myogenic effects of PDRN

Recent studies have introduced that PDRN, including its degraded components ATP and adenosine, supports myogenic differentiation of myoblasts by following signal pathways. Marco-Bonilla et al. [18] investigated the roles of overdosed extracellular ATP to support the myogenesis of C2C12 myoblasts by the activation of A2AR and A2BR. They demonstrated that the activation of adenosine A1A and A3A receptors (A1AR and A3AR) inhibits cyclic adenosine monophosphate (cAMP), and the activation of A2AR and A2BR promotes cAMP, which finally promotes myogenesis by up-regulation of the final effector phosphorylated cAMP response element-binding protein (CREB) in the AMP-activated protein kinase (AMPK)/cAMP pathway. In this study, the addition of tenofovir (ATP release inhibitor) and dipyridamole (adenosine reuptake inhibitor) was shown to modulate adenosine nucleotide concentrations and specific muscle differentiation markers. Tenofovir promotes premature muscle differentiation by reducing A2BR activation and AMPK signaling. Meanwhile, dipyridamole counteracts these effects by increasing extracellular adenosine, activating A2BR, enhancing AMP/ATP ratios, cAMP, and protein kinase A catalytic subunit alpha (PKAα) activation to support controlled myogenic differentiation, suggesting the roles of extracellular ATP and the AMPK/cAMP pathway in myogenesis.

Premature myoblasts colocalize neuronal nitric oxide synthase and nAChR, which help in the formation of multinucleated myotube precursors that regulate Ca^2+^ spikes for contraction and relaxation activities [19,20]. In line with this, Bernareggi et al. discovered that extracellular adenosine interacts with A2BR to activate the cAMP/PKA pathways, leading to the opening of nAChR and facilitating Ca^2+^ spiking contractions. The authors showed that blocking cAMP/PKA pathways with MRS 1754 (antagonist) impairs both nAChR function and muscle cell activity, suggesting that the cAMP-dependent pathway links A2BR activity to the regulation of nAChR properties, contributing to the electrical and mechanical processes critical for myogenesis [21]. Irrera et al. [7] investigated the dual roles of PDRN as an agonist for A2AR-mediated Wnt/β-catenin signaling modulation and an antagonist for NF-κB pathways. The authors showed that PDRN reduces the expression pro-inflammatory markers, such as phosphorylated NF-κB (pNF-κB), tumor necrosis factor-alpha (TNF-α), IL-6, and IL-12, through A2AR activation, as well as modulates immune responses by increasing interleukin-10 (IL-10) and Wnt/β-catenin expression. The suppression of NF-κB pathways can down-regulate the expression of pro-inflammatory cytokines and chemokines, suggesting a faster transition from the inflammatory phase to the regenerative phase for enhanced muscle regeneration [22,23]. Additionally, activation of the Wnt/β-catenin pathway is a well-known mechanism for myogenesis. Han et al. investigated the role of myoferlin (i.e., ferlin family protein that supports plasma membrane integrity, myoblast fusion, and vesicle trafficking [24]) as a regulator for the canonical Wnt/β-catenin signaling pathway [25]. Silencing myoferlin (MyoF) lowered the myogenic differentiation of C2C12 cells, disrupting the expression of key Wnt target genes (e.g., Lef1, c-Myc, and active β-catenin), suggesting the crucial role of the Wnt/β-catenin pathway in myogenesis. In addition, the Wnt/β-catenin pathway aids in the maturation of premature myofibers by facilitating the assembly of the cadherin/β-catenin/actin complex, of which signaling disruption results in muscle developmental defects [26]. Other studies revealed that PDRN inactivates the NF-κB pathway, thereby preventing the formation of the inactive β-catenin/forkhead box O (FOXO) complex and promoting the assembly of the β-catenin/T-cell factor (TCF) complex essential for muscle maturation [7,27]. Additionally, Ko et al. [28] revealed that PDRN supports the up-regulation of cAMP, PKA, and CREB and the down-regulation of MAPK and NF-κB, which are coincident with the discussed studies.

While studies on the direct interaction between PDRN and myoblasts are still limited, PDRN’s role can be linked to the regulation of various signaling pathways involved in myogenic differentiation. The literature in this section suggests that PDRN and its degraded component ATP or adenosine facilitate myogenesis and muscle regeneration by selectively interacting with P2XR, A2AR, A2BR, and nAChR, highlighting their potential to enhance the growth and myogenesis of cells.

### Skin regenerative effects of PDRN

The wound healing process is initialized with inflammation, followed by neovascularization, matrix synthesis, epithelialization, and finial restoration. In this process, PDRN and adenosine have significant boosting effects. A2AR and A2BR are key regulators of granulation tissue formation and skin regeneration by promoting angiogenesis, collagen synthesis, and extracellular matrix (ECM) remodeling [29]. To date, the most actively studied applications of PDRN are in wound healing and cosmetics. A2AR enhances the production of vascular endothelial growth factor (VEGF) and basic fibroblast growth factor [30] while suppressing thrombospondin-1 to support vascularization and modulates cAMP signaling to regulate fibroblast growth and collagen synthesis [31]. Additionally, A2AR–cAMP influences type I and III collagen (Col I and Col III) production via PKA and exchange protein directly activated by cAMP 2 (Epac2) while controlling fibroblast and collagen synthesis [32,33]. Hypoxia-driven A2BR up-regulation further enhances angiogenesis, and A2AR disruption leads to disorganized tissue, emphasizing its role in wound healing [34,35]. Adenine phosphoribosyltransferase (APRT) is the key enzyme to provide adenylate nucleotides using adenine. APRT promotes diabetic wound healing by converting adenine into AMP and supplementing cellular ATP through the purine salvage pathway. In this process, elevated AMP activates AMPK, enhancing glucose metabolism and ATP production while improving energy homeostasis and the proliferation of fibroblasts and keratinocytes, as well as mitigates the oxidative stress caused by reactive oxygen species (ROS) [36]. The anti-inflammatory effects of PDRN enable its wide uses in diabetic wound healing. The purinergic P2Y12 receptor (P2Y12R)–ADP pathway in the inflammatory cells modulates inflammation by shifting macrophages from pro-inflammatory (M1) to anti-inflammatory (M2) phenotypes, increasing IL-10 and IL-13 levels and promoting transforming growth factor-beta production, leading to accelerated diabetic wound healing [37]. Another report showed that ATP-activated P2Y2R enhanced the expression of VEGF, Col I, fibronectin, and α-smooth muscle actin in fibroblasts [38]. P2Y2R^−/−^ mice showed down-regulation of these factors, delaying wound closure and impairing fibroblast differentiation into myofibroblasts, highlighting the modulating effects of P2Y2R in the wound-healing process. The A2AR pathway is also involved in the activation of endothelial cells via the c-Ski and ERK/CREB pathway. c-Ski synergizes with activator protein-1 (AP-1) and STAT3 to increase VEGF expression, further promoting angiogenesis while also down-regulating the antiangiogenic factor thrombospondin-1, reinforcing A2AR’s proangiogenic effects in wound healing [39]. The results highlight crucial role of A2AR in skin regeneration, showing that the deficiency of A2AR leads to decreased c-Ski expression, impairing angiogenesis and delaying wound healing. These molecular interactions suggest that A2AR-mediated angiogenesis through c-Ski, STAT3, AP-1, and VEGF is essential for proper wound healing and could serve as a potential therapeutic target for improving angiogenesis-dependent skin regeneration. In summary, these studies collectively highlight adenosine signaling as a critical target for therapeutic modulation of skin regeneration and treatment of diabetic wounds.

### Osteogenic effects of PDRN

Extracellular adenosine signaling is a key metabolic pathway that regulates bone tissue formation, function, and homeostasis [40]. Adenosine regulates osteoblast and osteoclast activity, promoting bone formation while inhibiting excessive resorption. For example, the activation of ADORA2B enhances osteogenic differentiation of mesenchymal stem cells (MSCs), while ADORA2A reduces inflammation and improves bone healing by modulating immune responses [40]. Targeted adenosine delivery, including biomaterials that enhance its local availability, has shown promise in accelerating fracture healing and treating osteoporosis [41,42]. In particular, Mediero et al. [43] found that stimulation of A2AR directly with the agonist CGS21680 or indirectly by increasing endogenous adenosine levels with dipyridamole significantly enhanced bone regeneration. CGS21680 selectively binds to A2AR, leading to an increase in cAMP levels, which in turn activates PKA and downstream signaling pathways. The activation of A2AR suppressed osteoclast differentiation while enhancing the activity and function of mature osteoblasts, leading to the restoration of bone homeostasis following defect repair.

Additionally, the cAMP–PKA–ERK1/2 pathway has been found to suppress the nuclear translocation of NF-κB, thereby hindering the differentiation of osteoclasts [44]. Specifically, the activation of adenosine A2AR leads to increased cAMP levels, which activate PKA and subsequently phosphorylate ERK1/2. This activation inhibits the nuclear translocation of NF-κB by reducing inhibitor of kappa B (IκB) phosphorylation, thereby preventing its ubiquitination and degradation. As a result, NF-κB remains sequestered in the cytoplasm, leading to the inhibition of osteoclast differentiation. This pathway highlights the anti-inflammatory role of A2AR signaling in bone resorption and inflammatory diseases such as rheumatoid arthritis. On the other hand, Cheng et al. [45] reported that adenosine promotes osteogenesis in bone-marrow-derived mesenchymal stem cells (BMSCs) by activating STAT3 signaling, evidenced by increased phosphorylation and nuclear translocation of STAT3, leading to the up-regulation of osteoblast-specific markers such as alkaline phosphatase (ALP), Col I, Osterix, and Runt-related transcription factor 2 (RUNX2). This mechanism confirmed by using the STAT3 inhibitor S3I-201, which reduces calcium deposition and gene expression associated with osteogenic differentiation. Based on the mechanism, recent in vivo and in vitro studies found that PDRN can promote RUNX2 and collagen expression, calcium deposition, and osseointegration, highlighting its potential in bone tissue engineering [46–48].

## Application of PDRN for Tissue Regeneration

The use of bare PDRN in the clinic has several limitations. Firstly, PDRN is a chain of nucleotides, which has an overall negative charge due to its phosphate groups. This characteristic renders PDRN soluble in physiological solutions due to its hydrophilic properties, but it also indicates that the dispersion of PDRN in a solution makes it difficult for it to remain at a target site for a limited period. Secondly, since PDRN is exogenous DNA, it is recognized as foreign by the immune system. In general, it has been reported that 85% of PDRN is degraded within 120 min of exposure to the human body [5]. In order to utilize PDRN with these limitations for tissue regeneration, it is imperative that PDRN do not degrade and remain at the target site for a sufficient duration to allow tissue regeneration. Consequently, a cross-linked scaffold composed of biomaterials has been utilized for the purpose of stabilizing PDRN. The selection of the biomaterials to be combined with PDRN should be made on the basis of the biocompatibility, biodegradability, and mechanical properties of the target tissue.

### Application of PDRN for neuromuscular regeneration

There are many phenomena related to neural or muscular dysfunction. These specific statuses are particularly hazardous in that damage to neurons or muscles is likely to be irreversible. Nevertheless, a number of recent publications have sought to utilize PDRN for the purpose of regenerating neurons or muscles (Table S1). To properly deliver PDRN to target sites, it is also necessary to comprehend the mechanical characteristics of the specific organs of interest and the physicochemical and mechanical properties of the scaffold. The spinal cord, a representative model neuromuscular organ, exhibits mechanical properties that depend on geometric parameters: the cross-sectional area (CSA) of the spinal canal, the volume of cerebrospinal fluid, and the ratio of white matter to gray matter. These factors determine the mechanical properties of the spinal cord (Table S2). Tables S2 and S3 summarize the characteristics of the spinal cord as a representative neuromuscular organ, along with the biocompatibility, biodegradability, hydrophilicity or hydrophobicity, and mechanical properties of the scaffold used for regeneration.

#### PDRN for muscle tissue regeneration

**Fig. 2.**
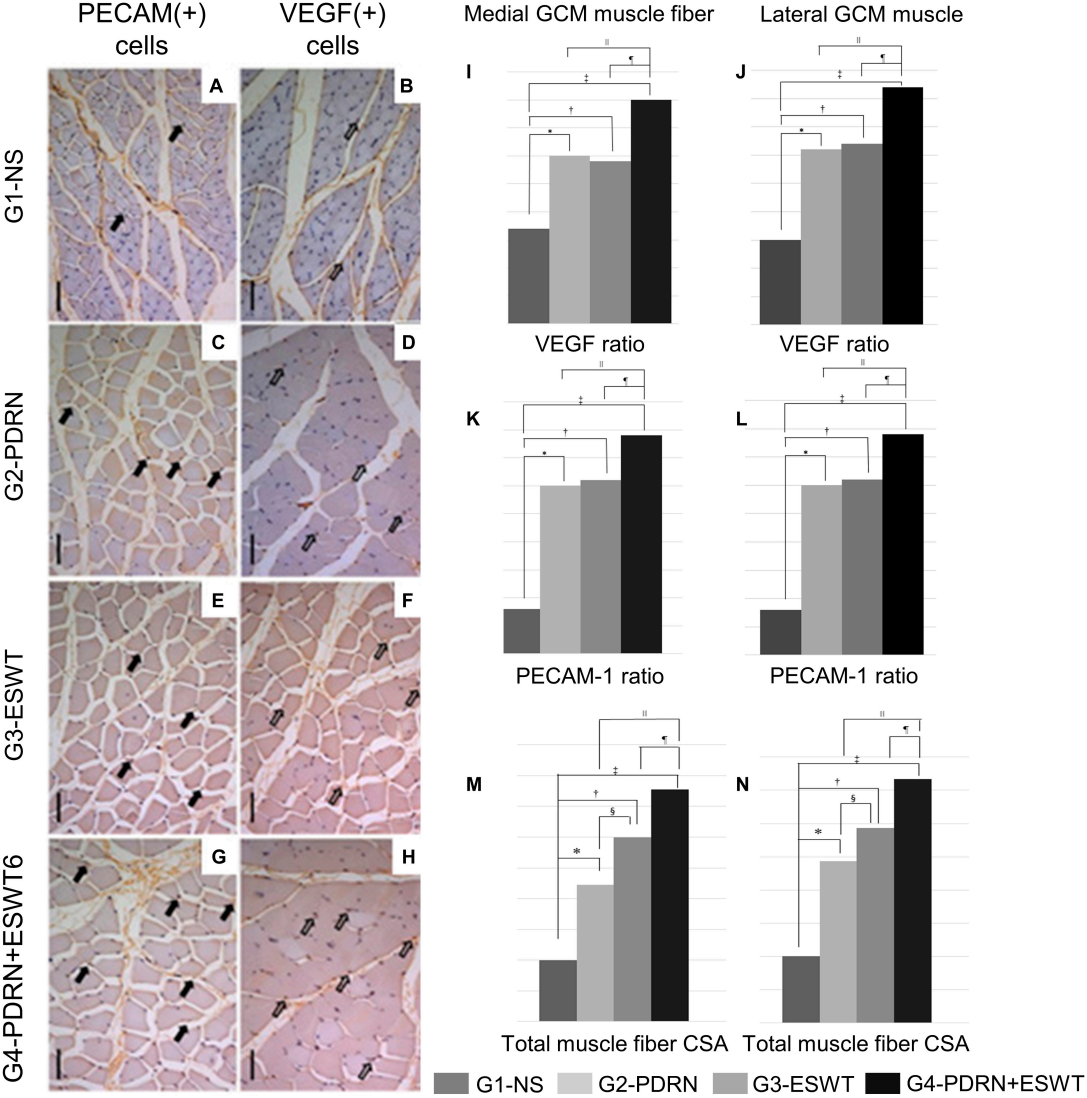
Immunohistochemical images and comparison of the mean cross-sectional areas (CSAs) of medial and later gastrocnemius (GCM) muscle fiber. (A to H) the VEGF and platelet endothelial cell adhesion molecule 1 (PECAM-1) ratios of the medial GCM muscle fibers in 4 groups. Those in group 4-PDRN with extracorporeal shock wave therapy (ESWT) (G4-PDRN+ESWT) were significantly higher than those in other 3 groups. (I and J) VEGF ratios in medial and lateral GCM muscle fibers, respectively. (K and L) PECAM ratios in medial and lateral GCM muscle fibers, respectively. (M and N) Total muscle fiber CSAs in medial and lateral GCM muscle fibers. Data reproduced with permission from Kim et al. [49]. Copyright AME Publishing Company 2022. G1-NS, group 1-normal saline; G2-PDRN, group 2-PDRN; G3-ESWT, group 3-ESWT. All symbols indicate *P* < 0.05 by one-way analysis of variance (ANOVA) with Tukey’s post hoc test (*group 1 vs. 2; ^†^group 1 vs. 3; ^‡^group 1 vs. 4; ^§^group 2 vs. 3; ^∥^group 2 vs. 4; ^¶^group 3 vs. 4).

One of the dysfunctions of the neuron and skeletal muscle status, atrophy, is defined as a reduction in muscle size to the extent that it becomes partially nonfunctional as a result of impaired nerve function. The underlying molecular biological causes of atrophy can be attributed to a reduction in myofibrillar protein, diminished metabolic enzyme function, alterations in the structure and function of blood vessels and nerves, and the accumulation of ectopic fat in skeletal muscles as a consequence of inadequate nutritional intake. Furthermore, there is an inability to regulate the nerves in target organs, as well as prolonged periods of immobilization. As shown in Fig. 2, Kwon’s group [49] employed radial extracorporeal shock wave therapy (ESWT) to facilitate the delivery of PDRN to relieve muscle atrophy by creating high pressure, which increased the probability of PDRN binding to A2AR on the muscle cell surface. Although radial ESWT has never been used to treat atrophied muscle, it is currently used in the clinical field to treat a variety of musculoskeletal problems, including rotator cuff, calcific tendinitis, lateral epicondylitis, stiffness, and plantar fasciitis. Furthermore, there are reports that it improves the function of sciatic nerves, alleviates denervation atrophy in a rat model, and is effective in the regeneration of skeletal muscle tissues after skeletal muscle injury by radial ESWT. Based on this effect of radial ESWT, it confirmed that the combination of radial ESWT and PDRN enhances angiogenesis and collagen synthesis to induce regeneration of skeletal muscle injury. In this study, 4 groups were prepared to observe the effects of PDRN with radial ESWT: group 1-normal saline (G1-NS), 0.7-ml injection of phosphate-buffered saline; group 2-PDRN (G2-PDRN), 0.7-ml injection of PDRN; group 3-ESWT (G3-ESWT); and group 4-PDRN with ESWT (G4-PDRN+ESWT), 0.7-ml PDRN injection with ESWT. They were administered normal saline or PDRN injections (0.7 ml, respectively) at 2 points, the lateral and the medial side of the gastrocnemius (GCM) muscle, under ultrasound guidance with a 5- to 13-MHz multifrequency linear transducer. To investigate their recovery, the right medial and lateral GCM muscle thicknesses, right calf circumference, compound muscle action potential amplitude of the right tibial nerve, CSAs of the type I medial and lateral GCM muscle fibers, and CSA of type II muscle fibers were observed. From a molecular biology perspective, the restoration of damaged muscle strength necessitates a reduction in connective tissue and an increase in the number of muscle fibers. To quantify this increase, the concentration of VEGF and platelet endothelial cell adhesion molecule 1 (PECAM-1) was examined. In other words, a higher expression level of VEGF in muscle tissue is indicative of an increase in the number of muscle fibers and PECAM-1 is also known to be involved in angiogenesis, which is necessary for healing and the regeneration of nutrients and immune cells. These data showed that the circumference of calf muscles, the thickness of the GCM muscle, the compound muscle action potential of the tibial nerve, and the CSAs of GCM muscle fiber types I and II in the G4-PDRN+ESWT group were significantly greater than those in the other groups and the VEGF and PECAM-1 ratios of GCM muscle fibers in the G4-PDRN+ESWT group were significantly higher than those in the other 3 groups. Given that both ESWT and PDRN have the capacity to enhance the expression of VEGF and PECAM-1, the data showed that the combination of these 2 factors exerted a synergistic effect (Fig. 2). Nevertheless, the optimal injection method and concentration for the restoration of atrophied muscles remain to be determined.

#### PDRN for neural tissue regeneration

Neurological dysfunctions such as spinal cord injury are serious disabilities that result in partial or complete sensory and motor dysfunction below the level of injury. To recover from this injury, enhancement of axon regeneration, neuron differentiation, and anti-inflammation is required. Thus, biomaterials with bioactivators and other materials to mimic the microenvironment of the disease site are used to modulate the inflammatory microenvironment, regulate the inhibitory microenvironment, and reshape the revascularization microenvironment.

**Fig. 3.**
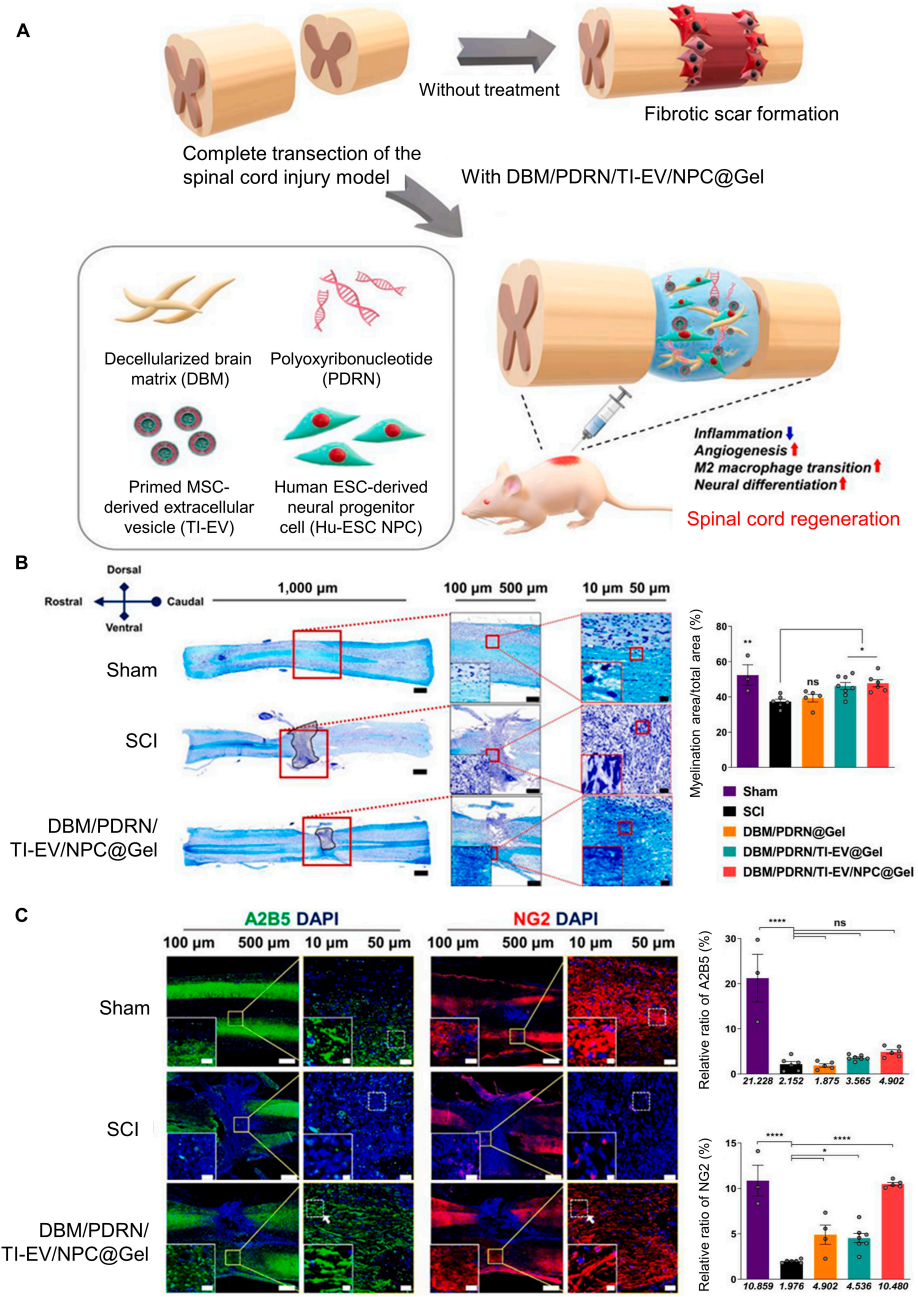
In vivo experiment for regeneration of the spinal cord. (A) Schematic illustration of a multimodal therapy strategy using a functional hydrogel. (B) Representative images of Luxol Fast Blue (LFB) staining 28 d post-DBM/PDRN/TI-EV/NPC@Gel injection in spinal cord injury (SCI). (C) Representative immunofluorescence images for the oligodendrocyte progenitor and neuroendocrine cell marker A2B5 (left) and the oligodendrocyte precursor cell surface marker neuron–glial antigen 2 (NG2) (right). Data reproduced with permission from Roh et al. [50]. Copyright AME Publishing Company 2022. ns, not significant; *P < 0.05, **P < 0.005, ***P < 0.001, and ****P < 0.0001. MSC, mesenchymal stem cell; ESC, embryonic stem cell; DAPI, 4′,6-diamidino-2-phenylindole.

As shown in Fig. 3, Roh et al. [50] conducted a grafting procedure involving a decellularized brain matrix (DBM), TNF-α/interferon-gamma-primed extracellular vesicles (EVs) derived from MSCs, and PDRN, which were then combined with a hydrogel in order to facilitate the treatment of spinal cord injury. The test groups consisting of DBM/PDRN@Gel, DBM/PDRN/TI-EV@Gel, DBM/PDRN/TI-EV/NPC@Gel, and the control group were prepared, and then the extent of neuronal regeneration and remyelination, along with the resulting motor functional recovery, was assessed 28 d following the administration of the treatment to the area of spinal cord injury. To demonstrate remyelination, the neuron–glial antigen 2 (NG2) marker for oligodendrocyte precursor cells [51] and the A2B5 marker for oligodendrocyte progenitor cells [52] were investigated to observe remyelination. The amount of the A2B5 marker showed no significant difference between the test group and control group. However, the amount of NG2 was significantly increased in the DBM/PDRN/TI-EV/NPC@Gel group, suggesting that oligodendrocyte progenitor cells were differentiated into oligodendrocyte precursors (fully or partially differentiated) to proceed with myelinization process because an oligodendrocyte precursor cell with an NG2 marker is a direct descendant of an oligodendrocyte progenitor cell with an A2B5 marker [53]. Furthermore, the data revealed that DBM/PDRN/TI-EV@Gel has multifunctional activity with angiogenesis, anti-inflammatory, and antiapoptotic effects, and encourages neurodifferentiation [50]. Despite the many advantages of DBM/PDRN/TI-EV@Gel, when implanted in in vivo experiments, the hyaluronic acid (HA) hydrogel employed in this study is a cross-linked formulation with a shape that is challenging to maintain in comparison to 3-dimensionally (3D) printed scaffolds (Fig. 3).

### Application of PDRN for skin regeneration and diabetic wound healing

PDRN is increasingly recognized for its role in skin regeneration and diabetic wound healing. It offers unique benefits in cellular repair and tissue regeneration [4]. For skin rejuvenation, PDRN and similar compounds like PN improve skin texture, elasticity, and hydration by promoting fibroblast activity, collagen synthesis, and angiogenesis, leading to visible antiaging effects [54]. Clinically, commercial formulations such as Rejuran have been widely utilized for skin revitalization and wrinkle reduction, with studies confirming their role in enhancing collagen deposition and remodeling. PDRN is used in various products for therapeutic purposes, such as Placentex, particularly popular in Italy and South Korea for diabetic wound healing and skin repair. Administered by healthcare professionals, PDRN-based formulations enhance wound closure, collagen production, and tissue regeneration, particularly in chronic or diabetic wounds.

While PDRN-based formulations have been commercialized, their standardization and regulatory approval processes remain complex. Further research is required to optimize biomaterial–PDRN interactions, degradation kinetics, and safety profiles, ensuring widespread clinical adoption in dermatology. Key challenges include determining optimal dosage regimens and delivery methods and ensuring long-term stability in formulations. Moreover, the potential side effects of PDRN treatments, such as immune responses or unintended angiogenesis, require further investigation to assess their long-term safety in clinical settings. The efficacy and side effect profiles of PDRN are also influenced by how it is integrated into various biomaterial matrices, such as hydrogels, which can impact stability and controlled release.

Meanwhile, in diabetic wound healing, PDRN is effective in stimulating tissue regeneration, angiogenesis, and collagen production, helping to enhance wound closure and reduce inflammation. Recent advancements in hydrogel-based delivery systems facilitate PDRN’s sustained release and noninvasive application, providing a promising approach for wound healing, particularly in challenging cases like diabetic wounds. Current research and ongoing clinical trials will help elucidate the optimal PDRN dosages, delivery mechanisms, and formulations that can provide the best therapeutic outcomes.

#### Skin regeneration and rejuvenation with PDRN or PN

PDRN and PN, both DNA-derived compounds, have shown significant potential in skin regeneration and rejuvenation due to their capacity to stimulate cellular repair and tissue regeneration (Table S4) [55,56]. By promoting fibroblast activity, collagen synthesis, and angiogenesis, PDRN and PN are effective in revitalizing skin, improving texture and elasticity, and reducing visible signs of aging, such as wrinkles and fine lines (Table S4) [57]. Most research on PDRN’s effects on skin regeneration has focused on fibroblast cell models [58,59]. However, physiological wound healing is a complex process involving interactions among inflammatory cells, fibroblasts, keratinocytes, endothelial cells, growth factors, and enzymes. PDRN not only enhances cell proliferation and migration but also promotes collagen accumulation, which is essential for counteracting collagen degradation and wrinkle formation during aging. Photoaging, a primary cause of wrinkles, is driven by collagen changes. At the same time, ultraviolet exposure induces matrix metalloproteinase (MMP) synthesis, leading to connective tissue damage through MMP-mediated collagen breakdown and subsequent skin aging [60]. Shin et al. [61] found that administering ERK inhibitors to fibroblasts increased MMP expression while reducing PDRN-mediated collagen accumulation, suggesting that PDRN may stimulate collagen production via ERK phosphorylation. In aging skin, inflammation reduces collagen activity and increases MMP levels, heightening the risk of skin cancers. HA fillers are commonly used in skin regeneration, but they may contain hyaluronan-associated proteins and 1,4-butanediol diglycidyl ether, which increase the risk of hypersensitivity reactions [62]. Small HA fragments may also trigger inflammation, leading to mild side effects such as erythema, swelling, hematomas, itching, or pain.

In response, alternative fillers, including new products synthesized with PDRN or PN extracted from salmon germ cells, have been developed. HA–PDRN and HA–PN fillers have a lower risk of side effects and are effective in filling skin spaces and promoting tissue regeneration [63,64]. When combined with HA, the rejuvenating effects of PDRN and PN are enhanced [65]. HA’s moisture-retaining properties allow deep skin hydration, providing a smoother, more youthful appearance. Together, PDRN, PN, and HA create a synergistic effect: HA improves hydration and elasticity, while PDRN and PN support cellular repair, elasticity, and structural integrity by stimulating collagen synthesis. This combination enhances skin firmness, minimizes fine lines, and produces an overall radiant, youthful glow.

**Fig. 4.**
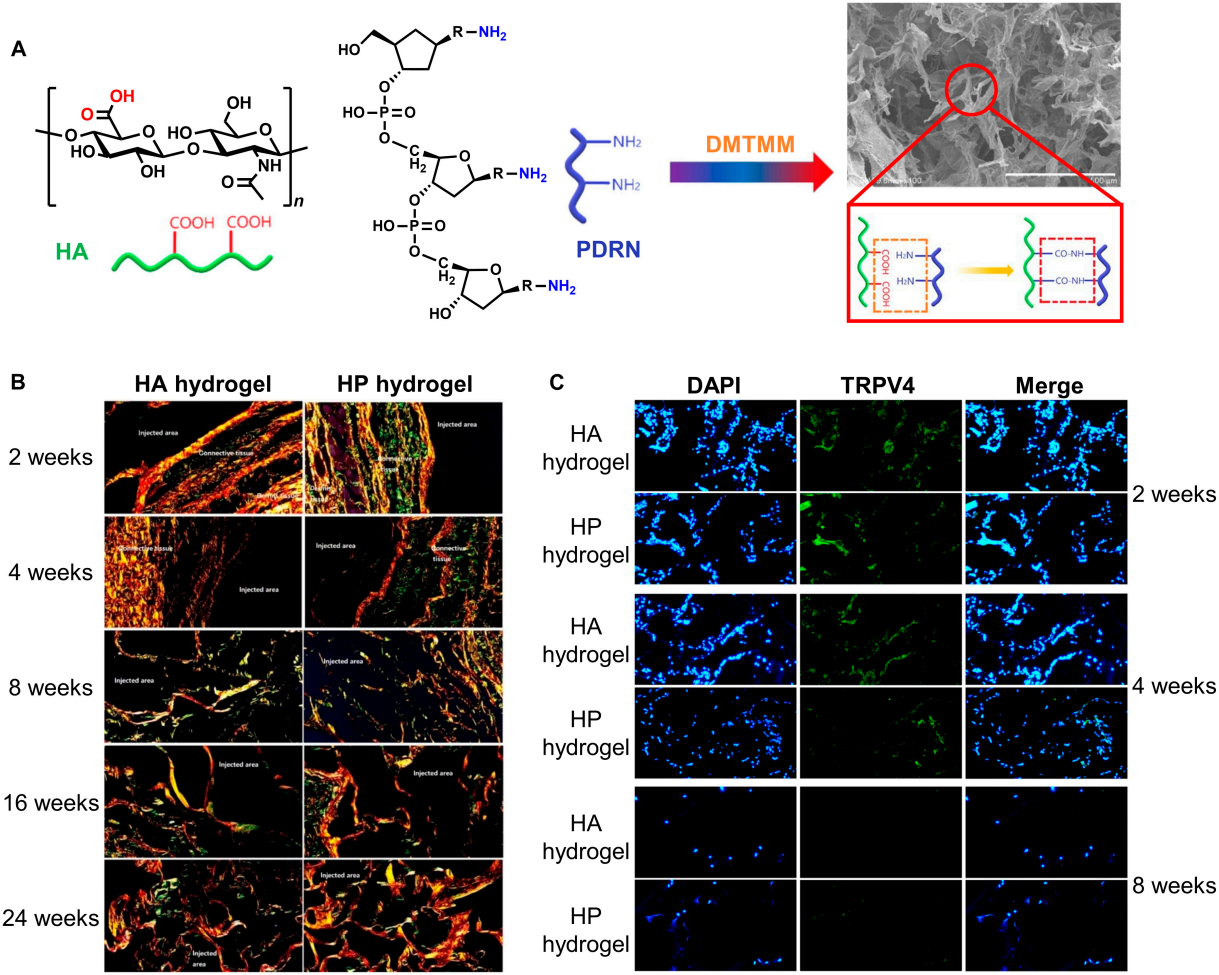
Hyaluronic acid (HA)–PDRN (HP) cross-linked hydrogel for skin regeneration. (A) Fabrication process and morphology of HP cross-linked hydrogel. (B) Assessment of collagen synthesis over time, visualized through Sirius Red staining, at 2, 4, 8, 16, and 24 weeks posttreatment with HA hydrogel and HP hydrogel. (C) Comparative analysis of transient receptor potential vanilloid 4 (TRPV4) expression in mouse tissue following treatment with HA hydrogel and HP hydrogel, evaluated using immunofluorescence staining. Data reproduced with permission from Guo et al. [63]. Copyright Elsevier 2024. DMTMM, 4-(4,6-dimethoxy-1,3,5-triazin-2-yl)-4-methylmorpholinium chloride.

Guo et al. [63] prepared an HA–PDRN (HP) cross-linked hydrogel via covalent interaction between the carboxyl groups of HA and the amino groups of PDRN in the presence of 4-(4,6-dimethoxy-1,3,5-triazin-2-yl)-4-methylmorpholinium chloride (DMTMM). (Fig. 4A). The optimized formulation, containing 0.6% HA and 0.5% PDRN, exhibited enhanced mechanical strength and stability due to increased cross-linking density, which contributed to its prolonged biodegradability in vivo. This formulation elicited no inflammatory response, while fibrosis encapsulation and angiogenesis gradually decreased over time, accompanied by increased fibroblast proliferation and ECM deposition, thereby reinforcing the hydrogel’s structural integrity (Fig. 4B). Compared to HA hydrogel, HP cross-linked hydrogel promoted collagen synthesis and strengthened collagen fiber bundles, effectively stimulating fibroblast growth for skin regeneration while providing enhanced volumetric stability and mechanical support. Furthermore, the incorporation of PDRN played a crucial role in modulating hydrogel degradation, ensuring controlled breakdown and prolonged bioactivity. Additionally, HP cross-linked hydrogel also down-regulated the expression of transient receptor potential vanilloid 4 (TRPV4), a calcium-permeable cation channel linked to MMP and inflammatory cytokine expression upon skin stimulation, thereby mitigating inflammatory responses and contributing to the inhibition of skin aging (Fig. 4C).

According to Kim et al. [64], an HA–PN complex filler demonstrated the highest proliferation rates for human fibroblasts when combined at 0.1% HA and 0.5% PN, outperforming HA or PN alone. This combination also stimulated greater collagen synthesis in both human and mouse fibroblasts, thereby contributing to improved skin regeneration. Moreover, the HA–PN complex demonstrated superior mechanical properties, including increased elasticity, viscosity, and viscoelasticity, which enhanced the filer’s structure integrity and in vivo stability. In addition, the HA–PN complex exhibited reduced TRPV4 expression and low levels of MMP and inflammatory cytokines, indicating diminished skin irritation and an improved capacity to inhibit skin aging relative to HA filler alone. While PDRN and PN are individually effective in skin regeneration, their combination with HA further boosts skin hydration, optimizes hydration and elasticity, enhances biomechanical stability, and prolongs biodegradability, establishing them as valuable tools in cosmetic dermatology.

#### Diabetic wound healing: Polysaccharide-based hydrogels with PDRN

PDRN has shown promising potential in treating diabetic wounds due to its ability to stimulate tissue regeneration, promote angiogenesis, enhance osteoblast activity, increase collagen synthesis, and reduce inflammation [3]. PDRN is particularly effective in diabetic wound healing through its stimulation of fibroblast proliferation, cell migration, and angiogenesis via A_2_A receptor activation and salvage pathways (Table S5) [5,66]. For example, Galeano et al. [67] found that daily PDRN injections in a diabetic (db/db) mouse model significantly increased the expression of VEGF and CD31, enhancing wound tensile strength compared to that of controls. However, due to PDRN’s short half-life (~3 h), daily intradermal or intramuscular injections are often required, which can be inconvenient for patients and deter treatment compliance [68]. This has led to the development of hydrogel-based delivery systems for more convenient and effective PDRN administration.

Hydrogels are advantageous in drug delivery and tissue engineering, as their 3-dimensional networks retain a significant amount of water and provide sustained, controlled release of water-soluble drugs, cells, proteins, and small molecules without degradation [69,70]. They are highly biocompatible, maintain a moist wound environment that promotes granulation tissue formation, and reduce pain, making them well suited for wound healing applications [71,72]. Common hydrogels for wound treatment include synthetic and natural polymers like alginate (Alg), HA, and chitosan derivatives [73,74]. Alg, in particular, has long been valued as a wound dressing material due to its excellent biocompatibility and unique topography [75,76]. Alg gels upon interaction with polycations (e.g., Ca^2+^ and Ba^2+^) and the sugar carboxyl group [77]. Shin et al. [78] presented Alg–PDRN hydrogels that allow sustained PDRN release, reducing the frequency of injections. An Alg–PDRN hydrogel showed enhanced cell proliferation and angiogenesis factor expression, significantly accelerating wound closure in diabetic mice over 14 d. Continuous PDRN release by the hydrogel promoted collagen deposition and advanced wound healing without requiring injection, showing promising potential as a noninvasive diabetic wound treatment. Similarly, double-cross-linked oxidized Alg (OA)–PDRN hydrogel combines ionic cross-linking and covalent interactions between the amino groups of PDRN and the aldehyde groups of OA [79]. In vivo, OA–PDRN hydrogels increased fibroblast proliferation, anti-inflammatory response, and ROS removal, providing an environment conducive to wound healing. In a diabetic Wistar rat model, OA–PDRN-treated wounds showed significant wound closure by day 10, complete regeneration by day 14, and higher vascularization than PDRN alone, due to sustained PDRN release and enhanced angiogenesis. Hydroxyproline levels, reflecting collagen synthesis, continued to increase in OA–PDRN-treated wounds, supporting the accelerated healing process. Chitosan is another promising natural polymer for hydrogel formation due to its high biocompatibility, moisture retention, and antimicrobial properties [80,81]. Chitosan–PDRN polyplexes support wound healing by promoting fibroblast proliferation and inhibiting bacterial biofilms, which can impede healing by altering the wound microenvironment [82,83]. The chitosan–PDRN combination showed enhanced antibacterial activity against *Escherichia coli* and *Staphylococcus aureus* and increased hydroxyproline content, suggesting significant collagen synthesis and wound healing. Additionally, CD31 expression increased, promoting angiogenesis, while inflammation markers like CD68 decreased, indicating effective tissue repair with minimized inflammation.

To further enhance PDRN’s therapeutic effectiveness, controlled-release hydrogels responsive to external stimuli like near-infrared (NIR) light are under investigation. Molybdenum disulfide (MoS_2_) nanosheets, which possess strong photothermal properties, convert NIR light into localized heat, thereby enhancing tissue penetration and minimizing potential side effects [84]. NIR-responsive hydrogels containing MoS_2_ nanosheets enable the on-demand release of PDRN, facilitating photothermal therapy to accelerate the healing process.

**Fig. 5.**
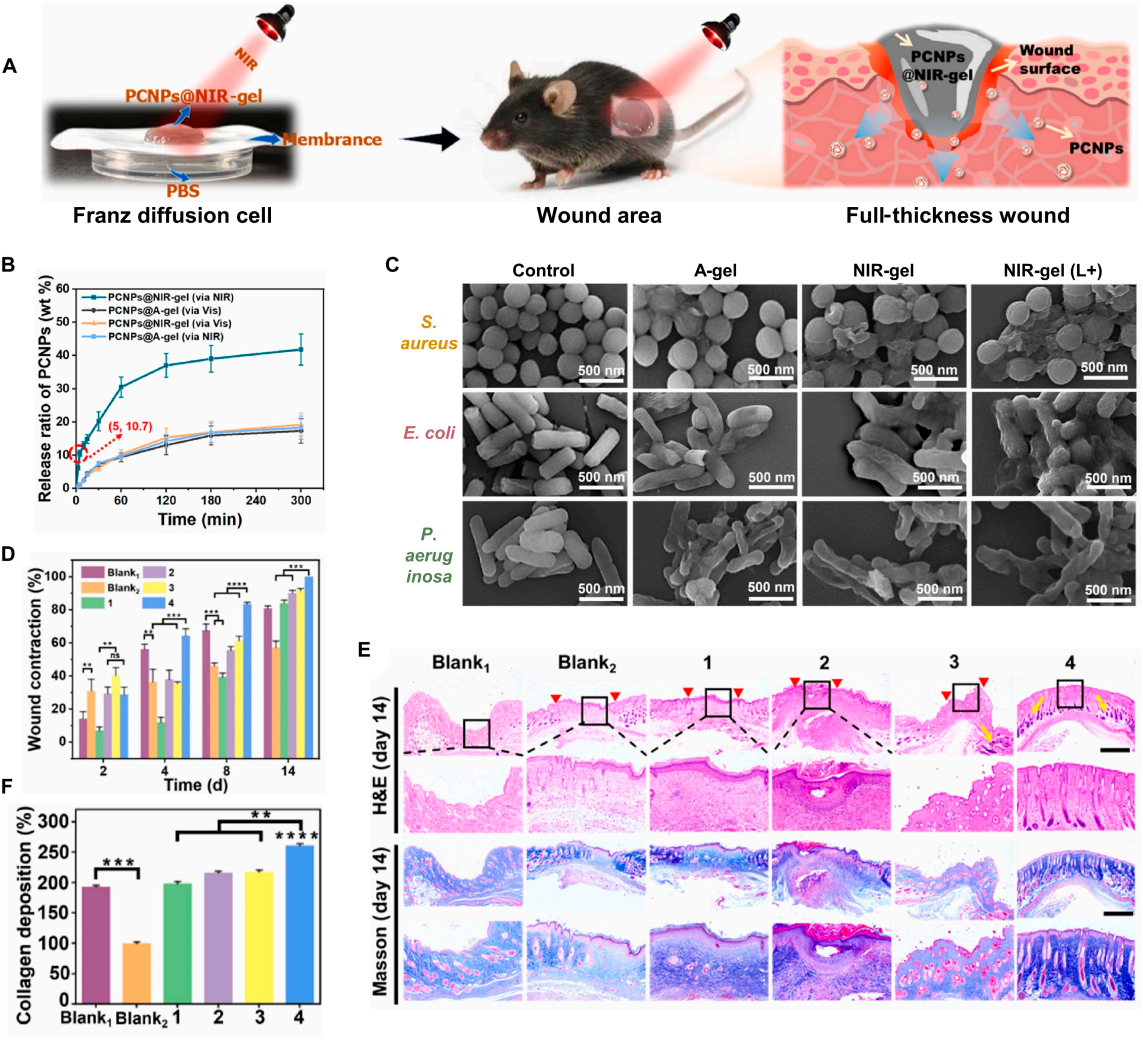
(A) Schematic of a skin wound model using a “Franz diffusion cell”-inspired apparatus. (B) PDRN release profiles for NIR-responsive nanocomposite hydrogel (PCNPs@NIR-gel) and PCNPs@A-gel under 5 min of continuous near-infrared (NIR) or visible (Vis) light exposure. (C) Scanning electron microscopy (SEM) images of *Staphylococcus aureus*, *Escherichia coli*, and *Pseudomonas aeruginosa* treated with A-gel, NIR-gel, and NIR-gel (L+). (D) Wound contraction rate in mice on days 2, 4, 8, and 14 posttreatment. (E) hematoxylin and eosin (H&E) and Masson’s trichrome staining of wounds across groups on day 14, with red arrows indicating the granulation tissue gap and yellow arrows indicating the skin epidermis. (F) Statistical analysis of the blue-stained area from Masson’s trichrome staining across groups. Statistical significance denoted as **P < 0.005, ***P < 0.001, and ****P < 0.0001. Labels: Blank_1_, nondiabetic mice; Blank_2_, treated with PBS; 1, treated with nucleic acid vectors (PCNPs); 2, treated with NIR-gel; 3, treated with NIR-gel (L+); 4, treated with PCNPs@NIR-gel (L+). Data reproduced with permission from Sun et al. [85]. Copyright Elsevier 2024. PBS, phosphate-buffered saline.

These hydrogels are engineered to provide stability to PDRN, protecting it from premature degradation before reaching the target site. Moreover, the mechanical strength of the hydrogel matrix, enhanced by MoS_2_ nanosheets, contributes to the structural integrity of the wound site, even under external stresses, thereby supporting tissue regeneration. For instance, the NIR-responsive nanocomposite hydrogel PCNPs@NIR-gel, which encapsulates MoS_2_ nanosheets alongside nucleic acid vectors (PCNPs), is synthesized through the copolymerization of quaternary chitosan and poly(*N*-isopropylacrylamide) (Fig. 5A) [85]. Upon exposure to NIR, the hydrogel undergoes temperature-induced shrinkage, triggering the controlled release of PDRN while promoting endothelial cell proliferation, migration, and VEGF expression (Fig. 5B). The incorporation of MoS_2_ nanosheets not only facilitates the release of PDRN but also contributes to the hydrogel’s biodegradability in vivo. The hydrogel’s gradual degradation over time ensures a sustained release of PDRN, maintaining its therapeutic effect throughout the healing process without causing long-term complications (Fig. 5B). When tested for antibacterial efficiency against *S. aureus*, *E. coli*, and *Pseudomonas aeruginosa*, the NIR-stimulated PCNPs@NIR-gel demonstrated significant bacterial cell wall damage (Fig. 5C). In a diabetic rat model, this hydrogel enhanced fibroblast proliferation, angiogenesis, and wound healing (Fig. 5D to F). The continuous presence of PDRN at the wound accelerated the repair process, while the NIR-triggered release system offered both efficient healing and infection control.

These findings underscore the synergistic interaction between PDRN and the biomaterials within the hydrogel, wherein the stability of PDRN is preserved, the mechanical properties of the hydrogel support tissue regeneration, and its biodegradability ensures the safe and effective long-term resolution of the wound. These PDRN hydrogel delivery systems—whether polysaccharide based for controlled release or NIR responsive with MoS_2_ nanosheets—represent innovative advancements for diabetic wound healing, effectively reducing inflammation, enhancing tissue repair, and providing an accessible, less invasive treatment option.

### Application of PDRN for bone regeneration

Bone is a specialized organ to support the construction of the human body. Based on their function, bones can be broadly divided into cortical bone and trabecular bone, each of which has different mechanical properties. These bones are susceptible to morphological changes and defects resulting from aging or disease. Additionally, they exhibit a deficiency in regenerative capacity, prompting the exploration of effective strategies to enhance bone regeneration, such as the utilization of PDRN with scaffolds. In order to effectively apply PDRN to bone regeneration, it is important to understand the mechanical properties of bone. Table S6 summarizes the characteristics of bone, as well as the biocompatibility, biodegradability, hydrophilicity or hydrophobicity, and mechanical properties of the scaffold used for bone regeneration (Table S7).

#### PDRN for bone regeneration in in vitro tests

Scandroglio’s group [2] studied the impact of PDRN at varying concentrations of fetal bovine serum (FBS) in vitro. They obtained human osteoblasts derived from a 5-year-old patient’s jawbone and then treated 100 μg/ml PDRN with 1%, 5%, and 10% FBS at 2, 4, and 6 d after seeding the cells. The results displayed that FBS was a critical element in modulating the impact of PDRN because the cell proliferation of human osteoblasts in 5% and 10% FBS media with PDRN showed a significant difference compared with the group without PDRN treatment. Furthermore, the researchers observed the mechanism of PDRN with DMPX, an A2 purinoceptor inhibitor, and suramine, a P2 purinoceptor inhibitor. In terms of cell proliferation, DMPX was observed to exert an inhibitory effect on cellular growth, with a reduction of 42.9% being noted. In contrast, suramine was found to have no discernible impact on cell growth. It was hypothesized that PDRN would utilize the A2R on the surface of a human osteoblast. However, there are some limitations to the applicability of PDRN in in vivo experiments because bones possess mechanical strength; therefore, PDRN should have to be attached to the site of the bone defect in order for sustained release of PDRN. To compensate for this limitation in previous research, Kim et al. [86] introduced a preliminary test to apply a scaffold consisting of poly(lactic-*co*-glycolic acid) polymer (PLGA, P), ricinoleic acid-modified magnesium hydroxide (mMH), and bone extracellular matrix (bECM, E) to an in vitro system. In this study, despite PLGA being a Food and Drug Administration-approved biocompatible material, it produces acidic by-products that generate inflammation in the in vivo environment [87,88]. Therefore, mMH was employed to neutralize the bone matrix that had been acidified by the by-product of PLGA [89,90]. The bECM, which is composed primarily of calcium and phosphate, was combined with PLGA and mMH with the objective of enhancing not only mechanical force (10.13 to 16.50 kPa), analogous to bone, but also the osteoconductivity of the scaffold [91]. PDRN was applied to the scaffold in order to enhance its osteoinductive properties, thereby facilitating accelerated bone formation. The alterations in the gene expression of the VEGF and MMP2 genes, which are involved in angiogenesis, as well as the IL-6 and IL-1β genes, which are associated with inflammation, were investigated following the seeding of human BMSCs. The results revealed that PDRN enhanced the expression of angiogenesis-related genes but suppressed the expression of inflammation-related genes. Furthermore, the ALP staining results, which are an early marker of osteogenesis, demonstrated that the PLGA (P) scaffold with magnesium hydroxide (M), bone-extracellular matrix (E), and PDRN (P) (PMEP) exhibited elevated ALP activity in comparison to those of the scaffold without PDRN. This suggests that PDRN may influence osteogenic differentiation of human BMSCs effectively [86].

#### PDRN for bone regeneration in in vivo tests

**Fig. 6.**
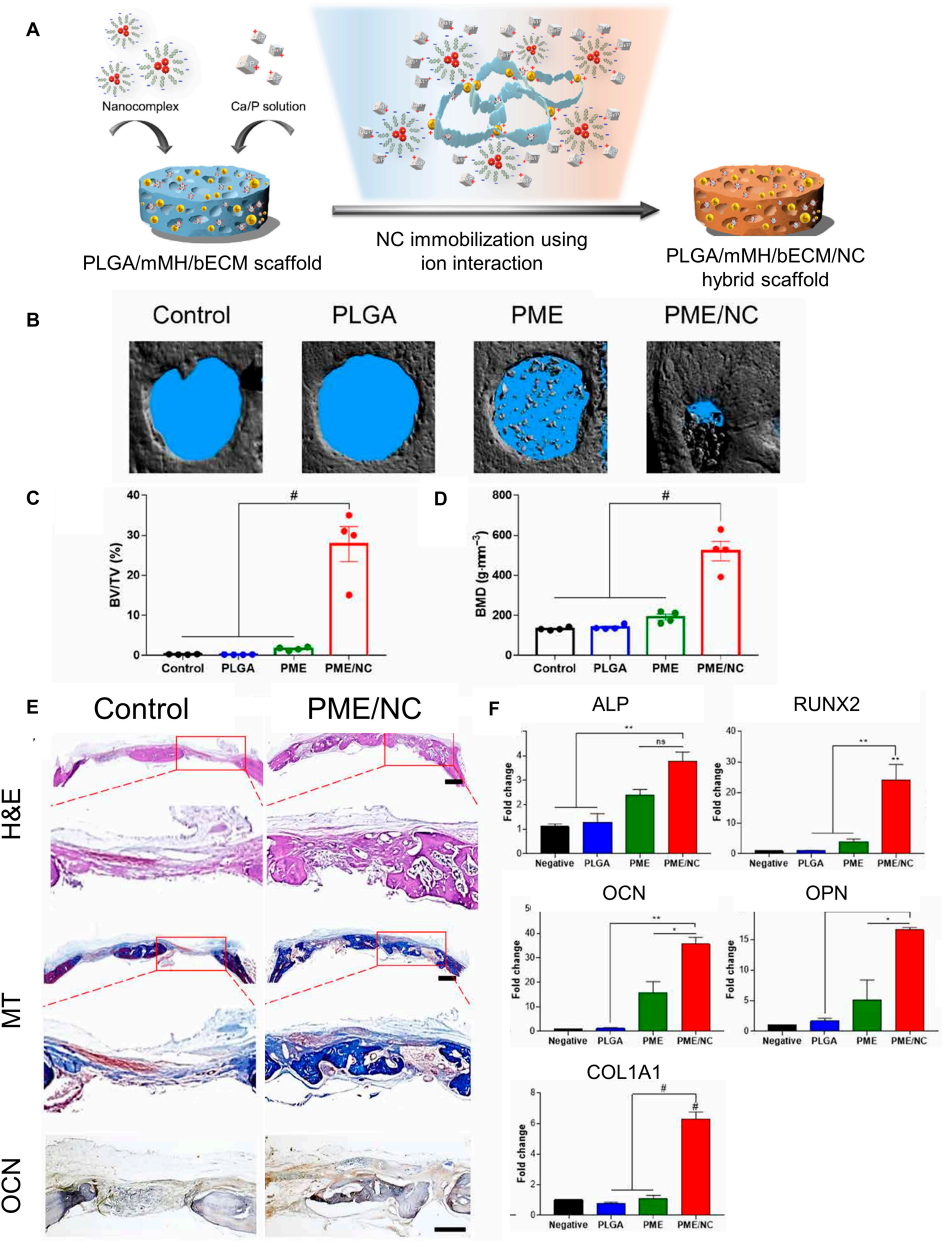
In vivo experiment for regeneration of bone. (A) Schematic illustration of the poly(lactic-*co*-glycolic acid) polymer/ricinoleic acid-modified magnesium hydroxide/bone extracellular matrix/nanocomplex (PLGA/mMH/bECM/NC) scaffold and PDRN/bone morphogenetic protein 2 (BMP2) NC preparation. (B) Representative 3-dimensional (3D) constructed images of a superficial view of the calvarial defect using micro-computed tomography (micro-CT). Quantified parameters of repaired bone, namely, (C) bone volume density (bone volume per total volume [BV/TV]) and (D) bone mineral density. (E) H&E, Masson’s trichrome (MT), and immunohistochemistry (IHC) analyses of antiosteocalcin after 8 weeks of implantation. Scale bars: 1,000 and 500 μm. (F) Expressions of genes related to osteogenesis: ALP, RUNX2, OCN, OPN, and COL1A1 (^#^*P* < 0.0001, ***P < 0.001, **P < 0.01, and *P < 0.05 indicate a statistically significant difference). Data reproduced with permission from Kim et al. [92]. Copyright American Association for the Advancement of Science (AAAS) 2021. BMD, bone mineral density; PME, PLGA (P) scaffold with magnesium hydroxide (M) and bone-extracellular matrix (E).

Recent advances in PDRN-incorporated scaffolds for bone regeneration are summarized (Table S8). Han’s group [86] published another research that developed the previous research on PDRN through the encapsulation of bone morphogenetic protein 2 (BMP2) and PDRN in this scaffold simultaneously and then implanting it in a calvarial defect model (Fig. 6) [92]. The primary advantage of this study is that the combination of 200 ng of BMP2 and PDRN yielded outcomes comparable to those observed with high concentrations of BMP2. This is because the conventional dose of BMP2 has the potential to stimulate excessive cell proliferation, which may result in adverse effects such as cancer development, ectopic bone formation, and renal problems [93].

In contrast to the approach taken by Han’s group, Byun’s group did not combine recombinant human bone morphogenic protein (rhBMP) and PDRN. Instead, they utilized a ceramic scaffold comprising hydroxyapatite and tricalcium phosphate, which exhibits stiffness analogous to the mechanical strength of bone, for encapsulation of 0.01, 0.05, or 0.1 mg/ml rhBMP and 0.1, 1, 5, or 10 mg/ml PDRN individually and then implanted it to the calvarial bone defect of a 7-week-old white rabbit. The radiological analysis revealed a significant difference between the group treated with 5 mg/ml PDRN and the control group in terms of bone formation at 8 weeks posttreatment. However, when histomorphometric analysis was employed to compare the 2 groups, no significant difference was observed. In contrast, the group treated with 0.05 mg/ml rhBMP-2 exhibited notable discrepancies in both assays. This research demonstrates that hydroxyapatite/tricalcium phosphate block scaffolds have sufficient mechanical strength and bone regeneration capacity when used with optimal concentrations of growth factors, but there are still limitations, which are the creation of a surgical wound at the donor site, longer operation time, discomfort at the donor site, and reoperation challenges if the bone graft fails [94]. Lee’s and Koo’s groups studied the effect of PDRN on early bone formation in beagle dogs undergoing implant placement with different surgical procedures. Lee’s group proceeded with the extraction of P2, P3, and P4 and then allowed alveolar ridges to heal for 2 months. The complex consisting of collagenated synthetic bone, which is commercially available, and PDRN was administered following a lateral-window sinus flow lift performed simultaneously with the surgical procedure [47,95]. Unlike Lee’s group, Koo’s group performed implant placement with bone graft, a procedure that was carried out with or without the incorporation of PDRN. Here, both groups employed the use of a collagen membrane because the incorporation of collagen into the bone substitute had been demonstrated to enhance stability and minimize the displacement of the bone graft during flap closure. Moreover, many research studies demonstrated that collagenated block bone has a function as a carrier for various growth factors or bioactive agents [96,97].

To investigate new bone (NB) formation in Lee’s group, augmented height, protruding height, and bone-to-implant contact in pristine bone were measured at 8 weeks after the surgical procedure. The composition of the augmented area, which was divided into 3 areas of interest (AOIs) located in coronal (C), middle (M) and apical (A) areas (A) (AOI_C, AOI_M, and AOI_A, respectively), was calculated with 3 parameters: the area percentage of new bone (pNB), residual bone graft particle (pRBP), and fibrovascular connective tissue (pFVT). However, the pNB, pRBP, and pFVT in AOI_A showed statistically significant differences between the control group and the test group. In the intragroup analysis, pNB and pFVT showed statistical differences within the control group (*P* = 0.001 and *P* = 0.003, respectively). pNB was significantly higher in AOI_C than in AOI_M and AOI_A (*P* = 0.014 and *P* < 0.001, respectively). pFVT was statistically significantly higher in AOI_A than in AOI_M (*P* = 0.001). No significant differences were found within the test group. In general, osteoprogenitor cells originate from pristine bone, especially the lateral window or sinus floor [98], meaning that NB formation decreases with increasing distance from clean bone. The results substantiate the osteoinductive potential of PDRN in inducing NB formation in regions that are unfavorable for regeneration, situated in proximity to the Schneiderian membrane and distant from the pristine bone. This phenomenon is particularly evident in the middle and apical regions [95].

Koo’s group transplanted collagenated biphasic calcium phosphate (CBCP) mixed with PDRN into male beagle dogs (aged 1 year) that had dehiscence defects, and they observed early stages of bone formation. Two teeth of the model animals were extracted, and 3-dimensional implants treated with either CBCP or CBCP/PDRN were placed in each group. The differences in bone formation between the groups treated with CBCP or CBCP/PDRN were quantified using the following parameters: bone volume per total volume, bone surface per bone volume, trabecular number, and structure model index. However, only the buccal augmented area showed significant differences in NB area and NB proportion, which may be up-regulated by PDRN to enhance oxygen and nutrients for angiogenesis. In terms of release kinetics, incorporating PDRN into block collagen bone grafts is more likely to result in a stable biological response due to less variability compared to that of powdered bone grafts. Therefore, the present preclinical study demonstrated that peri-implant NB formation was improved in the CBCP/PDRN group compared to that in the CBCP group in buccal augmented sites in a dehiscence defect model [47]. Ruggeri’s group [99] introduce heat-deproteinated bone (HDB) as a synthetic material candidate to replace it although autogenous bone is the preferred material for bone grafts. HDB is obtained by high-temperature thermal treatment; this process maintains the architecture of natural bone. Despite its good performance, a growth factor is required for osteoblast proliferation and differentiation. This group created a hole in the cortical bone and observed the rate at which the HDB/PDRN paste filled the gap by stimulating NB formation when compared with control groups treated with HDB or PDRN gel, respectively. While the test group treated with PDRN and HDB individually was able to observe natural healing in 12 weeks, the group treated with the HDB/PDRN paste was able to observe the hole filling in 4 weeks. Based on this observation, the advantage of the HDB material is that when the PDRN gel was treated alone, the PDRN could not be found in or around the hole due to dispersion of the PDRN gel. However, when it was treated with the HDB paste, the diffusion of PDRN was prevented, and it was supported to remain stable in the hole, thereby maximizing the effectiveness of PDRN [99].

## Conclusion and Future Perspectives

The remarkable pharmacological benefits and biosafety of PDRN in regenerative medicine have gained worldwide recognition. Being fragmented from DNA, PDRN minimizes immunogenicity, ensuring enhanced safety for clinical uses. Additionally, PDRN offers significant advantages as both a selective adenosine activator and a stimulator of the salvage pathway. As a selective A2AR agonist, PDRN avoids the side effects associated with nonselective adenosine activators such as adenosine and dipyridamole [5]. PDRN supplies nucleosides and nucleotides for tissue repair through the salvage pathway, promoting unique healing effects not shared by other DNA-derived drugs, making it a promising option for tissue regeneration. This paper extensively reviews the research progress of PDRN-based tissue engineering by categorizing its utilization in neuromuscular tissues, skins, and bones. Underlying Mechanism of PDRN for Tissue Regeneration outlines the biochemical mechanisms on how PDRN promotes tissue differentiation and regeneration. In Application of PDRN for Tissue Regeneration, we demonstrated that PDRN can be integrated or combined with various hydrogels, bioceramics, synthetic polymers, and photobiomodulation to enhance cellular behavior and differentiation into specific lineages, such as neurogenic, myogenic, osteogenic, and angiogenic ones. Each study was reviewed to explore the innovative combinations of PDRN with scaffold materials and their capacity to promote tissue regeneration and maturation from a mechanistic perspective.

In general, animals such as mice, rabbits, and dogs are most commonly used to study tissue regeneration using PDRN. A common method for them is to coat or encapsulate PDRN in a scaffold that takes into account the mechanical properties of each tissue and implant it directly into the tissue. In the case of bone, PDRNs are known to promote bone regeneration, but by 48 h after implantation, an inflammatory response occurs due to the activation of the immune system, which continues for 1 week. A significant increase in bone formation was observed only after a 2-week period had elapsed, in comparison to the control group. However, there are some limitations for research. Firstly, it is not easy to distinguish between bone graft and newly formed bone, and secondly, especially in humans, NB formation takes a long time (6 to 8 months) compared to that in animals. Therefore, selecting an appropriate animal model that can expedite this bone formation time is imperative to apply PDRN in the clinical field. In the case of neuromuscular regeneration, research on the optimal dose, number of injections, and formulation of the carrier, along with appropriate animal models, is still needed. In addition, it is necessary to continuously track the behavior of PDRNs coated or encapsulated in carriers to select the most suitable carrier to regenerate each tissue and improve the target efficiency of the PDRN.

Future research should include interdisciplinary studies and clinical trials to clarify the long-term efficacy and potential side effects of PDRN. Additionally, standardization and quality control of extraction processes are crucial to guarantee the safety, effectiveness, and consistency of PDRN-based regenerative medicines. In summary, researchers from various fields should work collaboratively to advance PDRN-based tissue engineering by tackling these challenges and implementing the recommended strategies.

## Data Availability

A data availability statement is not applicable to this article.

## Supplementary Materials

Supplementary 1 Tables S1 to S8 References [100–109] Graphical abstract
