# Supplementary material for: Versatile and Marvelous Potentials of Polydeoxyribonucleotide for Tissue Engineering and Regeneration
Source: Biomater Res. 2025 Apr 14;29:0183. doi: 10.34133/bmr.0183 (PMC11994882; doi:10.34133/bmr.0183)

**Front Matter**

Title

Versatile and marvelous potentials of polydeoxyribonucleotide for tissue engineering and regeneration

**Authors**

Nuri Oh^1^†, Juyoung Hwang^2,3,4^†, Moon Sung Kang^5^†, Chung-Yul Yoo^6^, Minseok Kwak^2,3,7^*, Dong-Wook Han^8,9^*

**Affiliations**

^1^ Department of Chemistry and Biology, Korea Science Academy of Korea Advanced Institute of Science and Technology, Busan 47162, Republic of Korea

^2^ Department of Chemistry, Pukyong National University, Busan 48513, Republic of Korea

^3^ Smart Gym-Based Translational Research Center for Active Senior’s Healthcare, Pukyong National University, Busan 48513, Republic of Korea

^4^ Ajou Energy Science Research Center, Ajou University, Suwon 16499, Republic of Korea

^5^ Research Institute of Mechanical Technology, Pusan National University, Busan 46241, Republic of Korea

^6^ Department of Energy Systems Research and Chemistry, Ajou University, Yeongtong-gu, Suwon 16499, Republic of Korea

^7^ Industry 4.0 Convergence Bionics Engineering, Pukyong National University, Busan 48513, Republic of Korea

^8^ Department of Cogno-Mechatronics Engineering, Pusan National University, Busan 46241, Republic of Korea

^9^ Institute of Nano-Bio Convergence, Pusan National University, Busan 46241, Republic of Korea.

**SUPPLEMENTARY MATERIALS**

***A. Supplementary table***

**Table S1.** Main composites and effects for neuromuscular regeneration with PDRN

| **Cell type/disease model** | **Materials (scaffold)** | **Surgical area** | **Concentration of PDRN or others** | **Salient outcomes** | **Ref.** |
| --- | --- | --- | --- | --- | --- |
| Atrophied calf muscles of the rabbits | PDRN with ESWT | Gastrocnemius muscles | 1.313 mg | - Cellular uptake of PDRN by ESWT ↑ - The protein expression of VEGF and PECAM-1 of GCM muscle fibers in G4-PDRN + ESWT ↑ | [49] |
| H9 hESCs/Rat spinal cord transection model | Hyaluronic acid (HA)-based hydrogel with  DBM/PDRN/TI-EV | T10 of spinal cord | 5 mg/mL DBM, 200 µg/mL | - Inflammation ↓ - Angiogenesis ↑ - M2 macrophage transition ↑ - Neural differentiation (NG2 marker) ↑ | [50] |

**Table S2.** Mechanical properties of spinal cord as neuromuscular organ

| Mechanical properties | White matter | Gray matter | Species | Ref |
| --- | --- | --- | --- | --- |
| Von Mises stresses (MPa) | 0.33 – 0.96 | 0.45 – 1.81 | Human | [105] |
| Compressive yield stress (kPa) | 80 ± 10.19 | 126.0 ± .4.4 | Porcine | [106] |
| Tensile yield stress (MPa) | 0.00005 – 0.05  0.01 – 0.05  1 – 1.5 | 0.0001 – 0.05  0.01 – 0.05  1.5 – 2.0 | Mice  Rabbit  Cow | [107-109] |

**Table S3.** The physicochemical and mechanical properties of the scaffold for neuromuscular organ

| Scaffold for PDRN | Biocompatibility | Degradability | Hydrophilic vs Hydrophobic | Mechanical properties | **Ref.** |
| --- | --- | --- | --- | --- | --- |
| Hyaluronic acid | Good | Biodegradable  Rate modifiable by crosslinking  (over 90% decomposed at 8 days) | Hydrophilic | - Compressive strength: 20 MPa - Pore size: 20-200 μm - Porosity ~90% | [110] |

**Table S4.** The strategy and effect of materials using PDRN or PN complex for skin regeneration

| **Cell type/**  **mouse model** | **Materials (scaffold)** | **Concentration of PDRN or others** | **Salient outcomes** | **Ref.** |
| --- | --- | --- | --- | --- |
| Normal human dermal fibroblast (HDF), 6-week-old mice | HP (HA–PDRN) crosslinked hydrogels | 0.5% PDRN, 0.6% HA | - excellent physiological stability and prolonged degradation - stimulates fibroblast growth for skin renewal - increases collagen fiber bundle thickness and higher proportion of type I collagen - TRPV4 protein expression ↓, MMP expression ↓, and inflammatory cytokine expression ↓ - supports volume enhancement when administered via a simple injection approach *in vivo* | [64] |
| Human skin fibroblast (HSF), mouse fibroblast (L929), 6-week-old SKH1 hairless female mice | HA–PN complex filler | 0.5% PN, 0.1 % HA | - fibroblast proliferation ↑ - collagen synthesis ↑, TRPV4 expression level *in vivo* | [65] |
| Dermal fibroblast from skin biopsies of young donor | Mixture of HA and PN | 7.5 mg/mL PN, 20 mg/mL HA | - skin fibroblast proliferation *in vitro* | [66] |

**Table S5.** The strategy and effect of materials using PDRN or PN complex for diabetic wound healing

| **Cell type/disease model** | **Materials (scaffold)** | **Concentration of PDRN or others** | **Salient outcomes** | **Ref.** |
| --- | --- | --- | --- | --- |
| HDF, diabetes mellitus fibroblasts, human umbilical vein endothelial cells (HUVEC), 8-weeks old C57BLKS/J-db/db male mice | PDRN-loaded alginate hydrogel (Alg-PDRN) | 100 μg/mL PDRN, 4 w/v% alginate | - FGF and VEGF quantity ↑, contributing to enhanced angiogenesis - synthesis of collagen, proliferation, and migration of fibroblasts ↑, VEGF expression level ↑ - collagen density increase in a diabetic mouse model - expression of TGF-β ↓, MPO ↓, α-SMA ↑ | [81] |
| HDF, HUVEC, RAW 264.7 cells,  Streptozotocin (STZ)-induced diabetic Wistar rats | Crosslinked PDRN-loaded oxidized alginate (OA) hydrogel | 4 mg/mL PDRN, 4 w/v% alginate | - HDF cell proliferation ↑ and migration capacity ↑ - expression of TNF-α ↓, IL-1β ↓, and IL-6 ↓ on RAW 264.7 macrophages - expression intensity of inflammation and angiogenesis-related chemokines ↓ - inflammation-related gene expression level ↓ | [82] |
| HDF,  9-weeks old STZ-induced diabetic Wistar rats | chitosan/PDRN polyplex | 1 mg/mL PDRN, 6 mg/mL chitosan | - cell proliferation ↑ and cell migration ↑ of HDF cells - promotes antimicrobial activity against both *E. coli* and *S. aureus* - enhances diabetic wound healing effect by providing sustained exposure to PDRN at the wound site - hydroxyproline content ↑ in the regenerated tissues of the wound - CD31 ↑ and CD68 ↓ expression on angiogenesis and inflammation | [85] |
| L929, NIH 3T3, 6-weeks old STZ-induced C57BL/6N male mice | An NIR-responsive hydrogel loaded with PDRN | 4 mg/mL PDRN, 70 μg/mL PCNP, 5 mg MoS_2_ | - control of the release behavior of PCNPs under NIR laser irradiation - promotes surface antibacterial activity against *S. aureus*, *E. coli*, and *P. aeruginosa* - accelerated fibroblasts migration in response to NIR stimulation - promotes epidermal regeneration, increases collagen deposition, accelerates angiogenesis, and exerts anti-inflammatory effects | [88] |

**Table S6.** Mechanical properties of bone

| Mechanical properties | Cortical bone | Trabecular bone | Species | Ref |
| --- | --- | --- | --- | --- |
| A porosity | 5 – 15% | 40 – 95% | Human | [111] |
| Tensile yield stress (MPa) | 71.56 ± 10.19 | 7.6 ± 2.20 | Human |  |
| Compressive yield stress (MPa) | 100.06 ± 16.36 | 10.4 ± 3.20 | Human |  |
| Shear modulus (GPa) | 4959 | - | Human |  |

**Table S7.** The physicochemical and mechanical properties of the scaffold for bone

| Scaffold for PDRN | Biocompatibility | Degradability | Hydrophilic vs Hydrophobic | Mechanical properties | **Ref.** |
| --- | --- | --- | --- | --- | --- |
| Poly(lactic-co-glycolic) acid  (PLGA) | Good | Biodegradable  Rate modifiable by copolymer composition  (over 90% decomposed at 14 days) | Hydrophobic  Hydrophobicity adjustable by copolymer composition | - Compressive strength: 1.31 – 1.43 Gpa - Porosity: 77 - 97% - Pore size: 200-300 μm | [89, 112, 113] |
| Gelation Methacrylate (GelMA) | High | Biodegradable  Rate modifiable by crosslinking  (over 90% decomposed at 28 days) | Hydrophilic | - Compressive strength: 0.75 – 1.38 MPa - Porosity: ~95% - Pore size: 100 - 200 μm | [114] |
| Hydroxyapatite (HA)/tricalcium phosphate (TCP) | High | Non-degradable, suitable for bone grafts | Hydrophilic | - Compressive strength: 6.76 Mpa - Porosity ~80% - Pore size: 200-400 μm | [98] |
| biphasic calcium phosphate (BCP) with collagen | High | Non-degradable, suitable for bone grafts | Hydrophilic | - Compressive strength: 4.32 Mpa - Porosity ~80%, - Pore size: 100 -600 μm | [99] |

**Table S8.** Main composites and effects for bone regeneration with PDRN

| **Cell type/disease model** | **Materials (scaffold)** | **Surgical area** | **Concentration of PDRN or others** | **Salient outcomes** | **Ref.** |
| --- | --- | --- | --- | --- | --- |
| Human osteoblast from jawbone  (5-year-old patient) | - | - | 100 µg/mL PDRN | - Osteoblast proliferation ↑ - Partially inhibit cell growth by DMPX - Not inhibit the cell growth by suramine | [89] |
| hBMSCs | PLGA/mMH/  bECM/PDRN | - | 100 µg/mL PDRN | - IL-6 and IL-1β for inflammation ↓ - VEGF and MMP2 gene expression for angiogenesis at 7 and 21 days ↑ - ALP, RUNX2, OCN gene expression for osteogenesis at 7 and 21 days ↑ | [90] |
| Human fetal derived MSC/ calvarial bone defect 8 weeks old Wistar rat | PLGA/mMH/  bECM/NC (PDRN and BMP2 nanocomplex) | Calvarium | 200 ng/scaffold BMP2 and PDRN | - Mechanical strength: 1.0 - 1.5 MPa - New bone formation observed 8 weeks post-treatment - VEGF and MMP2 gene expression for angiogenesis ↑ - ALP, RUNX2, OCN, OPN, and COL1A1 gene expression for osteogenesis ↑ - Bone mineralization ↑ | [96] |
| Calvarial bone defect 7-week-old white rabbit | HA/TCP (6:4) scaffold with Ca/P (1.5-2.0) | Calvarium | 0.1, 1, 5, 10 mg/mL PDRN/0.01, 0.05, 0.1 mg/mL rhBMP2 | - Mechanical strength: 6.76 MPa, Dimension: outer dimension 7 mm diameter x 5 mm height   Porosity ~80%, pore size was 200-400 μm   - New bone formation of 5 mg/mL PDRN treatment group and 0.05 and 0.1 mg/mL of rhBMP2 treatment group observed 8 weeks post-treatment | [98] |
| Male beagle dogs  (1-year-old) | Collagenated synthetic bone | P2, P3, P4 in maxilla | 1.875 mg/mL PDRN | - BICa, pNB, pRBP, and pFVT in only apical area of sinus ↑ | [99] |
| Male beagle dogs  (1-year-old) | Collagenated biphasic calcium phosphate (CBCP) with collagen membrane | P2, P3, P4 in maxilla | 1.875 mg/mL PDRN | - Stability of bone substitutes with collagen membrane ↑ - The displacement of bone graft material during fap closure with collagen membrane ↓ - NB area and NB proportion in the buccal augmented area at 2 and 6 weeks ↑ | [100] |
| Sprague–Dawley male rats | Heat-deproteinated bone (HDB) | Cortical bone | 1 mg HDB/  1 ml PDRN gel | - The paste form of PDRN gel/HDB prepared - The rate of new bone formation of the paste form ↑ | [104] |


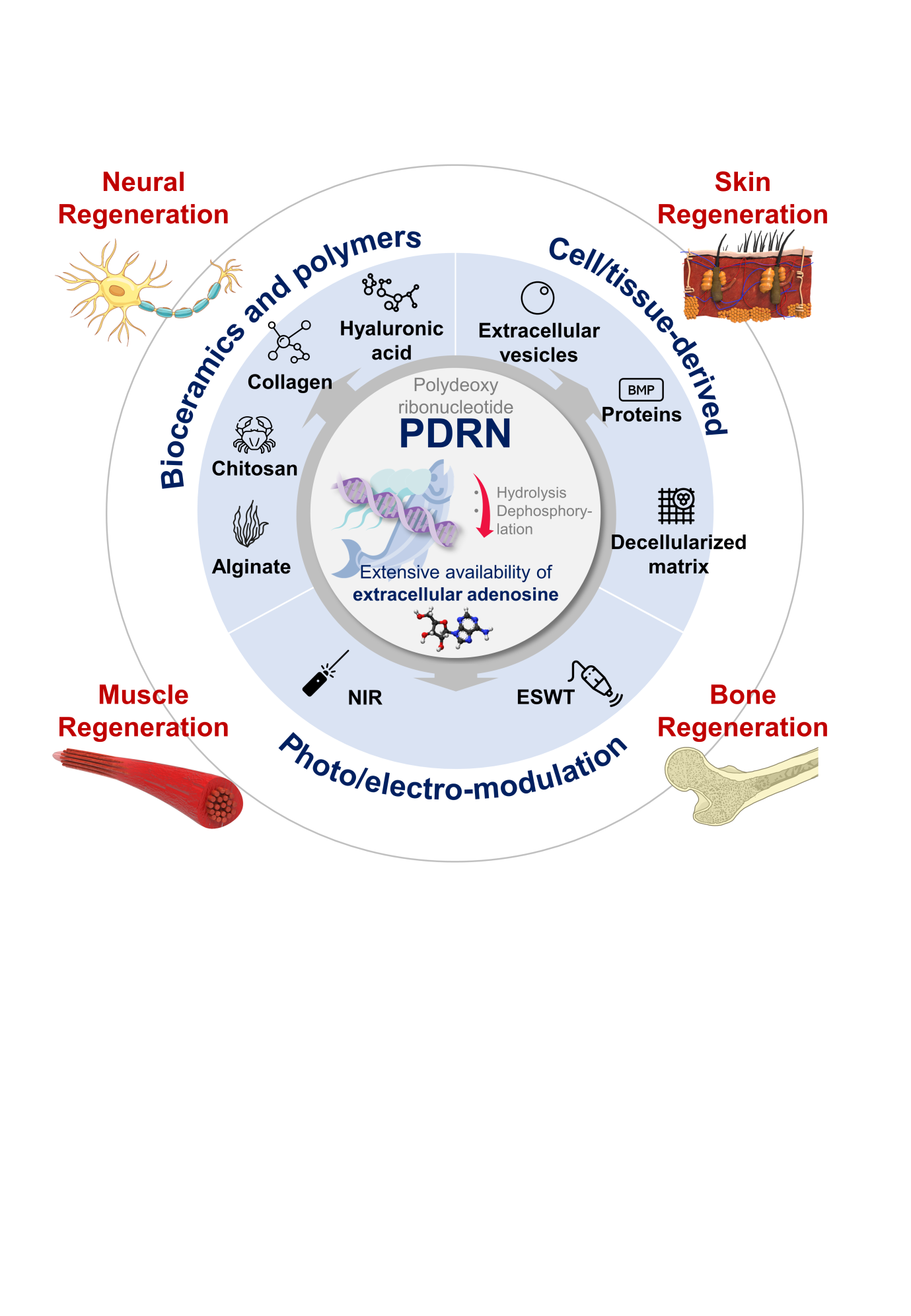

Supplement: Supplementary 1 — Tables S1 to S8 References [100–109] Graphical abstract [file bmr.0183.f1.docx]
